# Mutations in NYX of individuals with high myopia, but without night blindness

**Published:** 2007-03-01

**Authors:** Qingjiong Zhang, Xueshan Xiao, Shiqiang Li, Xiaoyun Jia, Zhikuan Yang, Shizhou Huang, Rafael C. Caruso, Tianqin Guan, Yuri Sergeev, Xiangming Guo, J. Fielding Hejtmancik

**Affiliations:** 1 State Key Laboratory of Ophthalmology, Zhongshan Ophthalmic Center, Sun Yat-sen University, Guangzhou, China; 2 Ophthalmic Genetics and Visual Function Branch, National Eye Institute, National Institutes of Health, Bethesda, MD

## Abstract

**Purpose:**

High myopia is a common genetic variant that severely affects vision. Genes responsible for myopia without linked additional functional defects have not been identified. Mutations in the nyctalopin gene (NYX) located at Xp11.4 are responsible for a complete form of congenital stationary night blindness (CSNB1). High myopia is usually observed in patients with CSNB1. This study was designed to test the possibility that mutations in the NYX gene might cause high myopia without congenital stationary night blindness (CSNB).

**Methods:**

The genomic sequence of NYX in 52 male probands with high myopia but without CSNB was analyzed through direct DNA sequencing. Variations in the NYX were verified by analyzing available family members and 232 controls.

**Results:**

Two unrelated male individuals with high myopia but without night blindness were found to have novel Cys48Trp and Arg191Gln mutations in NYX. The mutations were found to be located in distinct regions, different from the locations of mutations known to cause congenital stationary night blindness with myopia (CSNB1).

**Conclusions:**

Mutations in NYX may cause high myopia without CSNB. The observations suggest that NYX may have independent effects on myopia and night blindness.

## Introduction

Myopia, the most common visual defect, affects about 30% (3%-84%) of people [1-4]. Its extreme form, high myopia, affects 1%-2% of the total population. It is the fourth most common cause of blindness because of its association with vision-threatening conditions such as chorioretinal degeneration, retinal detachment and glaucoma. High myopia can be inherited as an autosomal dominant, autosomal recessive, X-linked recessive or possibly complex trait. Most high myopia is simple (non-syndromic) without other accompanied ocular or systemic abnormalities. No genes responsible for non-syndromic high myopia have been identified although several chromosome loci have been assigned [5-12]. Syndromic high myopia has been observed in Cohen syndrome (OMIM 216550), Knoblock syndrome (OMIM 267750), Stickler syndrome (type 1, OMIM 108300; type 2, OMIM 604841), Marfan syndrome (OMIM 154700), RP2 (OMIM 312600), and CSNB1 (OMIM 310500). Of these, CSNB1 is the only disease with functional defects without gross structural abnormalities. Several genes causing syndromes including high myopia have been identified, including COH1, COL18A1, COL2A1, COL11A1, FBN1, BFSP2, RP2, and NYX [13-21]. However, no mutation in any of these genes has been shown to cause isolated high myopia.

The human NYX gene (OMIM 300278) encodes a small leucine-rich protein (nyctalopin) with 481 amino acid residues [18,19,22]. The exact function of nyctalopin is still unknown. Mutations in NYX have been reported to cause congenital stationary night blindness (CSNB1) [18,19,23-28]. Patients described with CSNB1 usually have myopia (OMIM 310500). Phenotype analysis of patients with mutations in the NYX gene further documented that myopia in these patients is associated with the complete form of CSNB1 [28-30]. While mutations in the NYX gene might alter eyeball growth secondarily to their effects on rod function and vision, they might also be associated with refraction directly in addition to their effect on night vision. This suggests the possibility that some mutations in NYX could cause isolated myopia without night blindness, making it a reasonable candidate gene for isolated X-linked myopia. We report here novel mutations in the NYX genes in two unrelated male individuals with myopia alone without night blindness.

## Methods

### Patients with high myopia

Unrelated probands with high myopia were collected from the Zhongshan Ophthalmic Center as part of a project to identify genetic loci and genes for high myopia. Informed consent conforming to the tenets of the Declaration of Helsinki was obtained from the participating individuals prior to the study and the study was approved by the Institutional Review Boards of the Zhongshan Ophthalmic Center and the National Eye Institute. The diagnostic criteria for high myopia were basically as previously described [12]: (1) Myopia was noted before school age; (2a) bilateral cycloplegic refraction of -6.00D or lower (spherical equivalent) in individuals <30 years of age, or (2b) manifest refraction of -6.00D or lower (spherical equivalent) in individuals 30 years or more of age; and (3) Exclusion of other known ocular or systemic diseases. Standard ophthalmological examinations including visual acuity, slit-lamp and funduscopic examinations on all subjects and ERG, A-scan, B-scan, and fundus photography on selected patients were performed by ophthalmologists. Electroretinogram (ERG) responses were recorded according to ISCEV standards [31]. Genomic DNA was collected from 316 unrelated probands with high myopia. Any available first-degree family members were enrolled in the study if they agreed to participate. Of these families, 52 male probands were enrolled in the current study, after exclusion of those families in which high myopia is inherited as an autosomal trait by examination of the pedigree. Thus, while not all individuals included in the study necessarily have X-linked high myopia, the probands will be enriched for this group in so far as families with identifiable autosomal inheritance patterns have been excluded.

### Mutation screening of candidate genes

DNA fragments encompassing the coding exons and the adjacent intronic sequence in the NYX gene (NCBI human genome build 35.1, NC_000023 for genomic DNA, NM_022567 for mRNA, NP_072089 for protein) of the 52 unrelated probands with high myopia were amplified by polymerase chain reaction, using five pairs of primers as previously described [28].

DNA was sequenced using an ABI BigDye Terminator cycle sequencing kit v3.1 and electrophoresed on an ABI 3100 Genetic Analyzer. Variations in NYX were verified by analyzing 232 control individuals as well as family members for whom genomic DNA was available. The controls were of Chinese origin, living in Southern China.

### Molecular modeling

The model of the nyctalopin gene wild type protein (nyc) was built by homology modeling based on crystal coordinates for the Nogo receptor ectodomain Brookhaven protein database (PDB) [32] file: 1ozn.pdb as the structural template. The primary sequences of nyctalopin and 1ozn were aligned by the method of Needleman and Wunch [33], incorporated in the program Look, version 3.5.2 (Lee, 1993) for 3-dimensional structure prediction. Finally, the monomeric nyc was built using the automatic segment matching method in the program Look [34] followed by 500 cycles of energy minimization. The same program was used to generate conformation of genetic point mutations C48W and R191Q, and then to refine it by self-consistent ensemble optimization [35], which applies statistical mechanical mean-force approximation iteratively to achieve the global energy minimum structure. The geometry of the predicted structures was tested using the program Procheck [36].

## Results & Discussion

**Figure 1:**
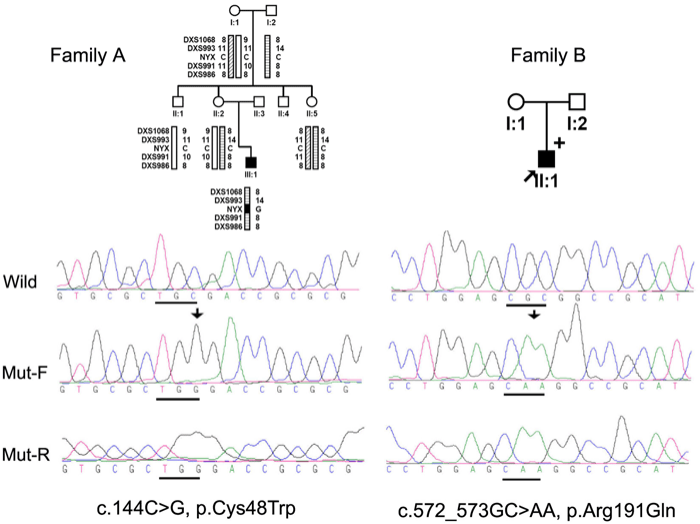
Pedigrees, haplotyes, and sequencing results of the two families with high myopia and NYX mutations. Filled squares represent individuals affected with high myopia. Haplotype analysis in Family A demonstrated that the c.144C>G mutation in III:1 is a de novo mutation. +: Mutation detected in II:1 of family B. Mut-F or Mut-R: Forward or Reverse sequencing of NYX gene fragments for individual III:1 in family A (left column) and individual II:1 in family B (right column). Wild: Forward sequencing of the corresponding fragments in normal control.

Mutations in NYX, c.144C>G and c.572-573GC>AA, were identified in 2 of the 52 probands, respectively. A de novo c.144C>G mutation was found in individual III:1 in family A (Figure 1). At the time of this study, the proband was 8 years old, with a prescription of -12D in both eyes with a history of having developed high myopia over -6D by 2 years of age. There are no signs of night blindness, and the patient and his family gave a history of normal vision at night, including being able to walk under dim light or walk upstairs or downstairs at night without stairway lights, just as well as his parents. Both his parents had normal vision, including night vision and neither was myopic. An ERG is not available for this individual and he and his family are not available for further examination. The corrected visual acuity is 20/40. There was neither nystagmus nor indication of abnormal color vision. The refraction for other family members examined in family A was between +1D to -1.5D. This mutation was not detected in any other family member (Figure 1, family A).

**Figure 2:**
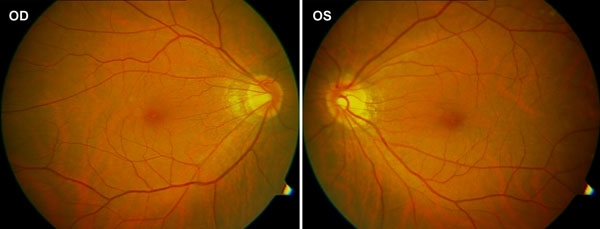
Fundus photographs. Typical changes of high myopia, including optic nerve head crescent and "tigroid" appearance of the posterior retina, were demonstrated.

**Figure 3:**
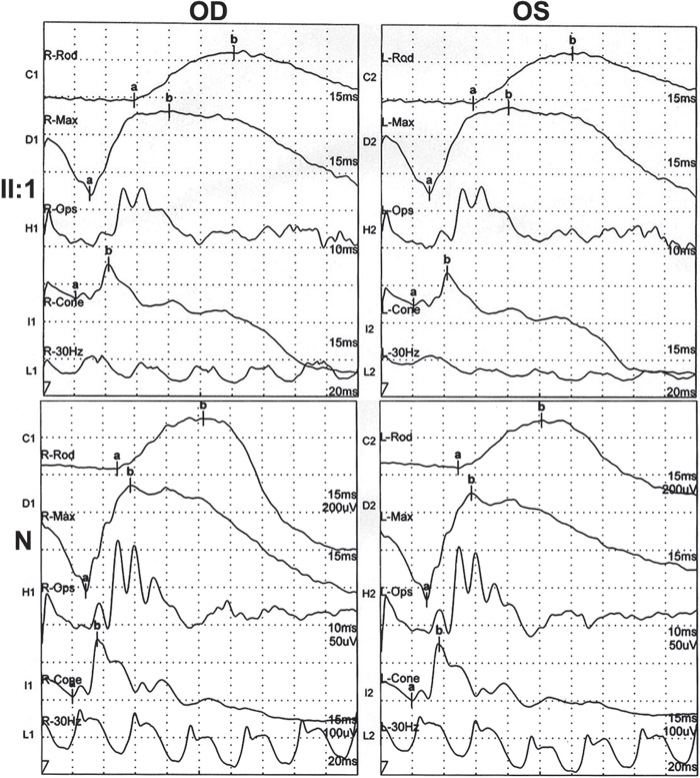
ERG recordings of individual II:1 in family B and that of a representative control (N). Normal rod responses and mild reduced cone responses were shown. Scale for each subdivision: C1, C2: 200uV; D1, D2: 200uV; H1, H2: 50uV; I1, I2: 100uV; L1, L2: 100uV.

A c.572-573GC>AA mutation was found in a 46 year-old man (Figure 1, II:1 in family B) with myopia of -6.50D bilaterally. He was first diagnosed with high myopia at the age of 40, although the condition might well have been present before that. His phenotype has been stable since that time. His corrected visual acuity was 20/20 at a recent examination. Ophthalmological examination revealed fundus changes typical of high myopia including an optic nerve head crescent and a "tigroid" appearance of the posterior retina, but no signs of retinal degeneration (Figure 2). Nystagmus was not observed and there was normal color vision. He has no difficulty walking under dim light as compared with his parents and friends. ERGs under scotopic and photopic conditions demonstrate a normal rod response and mildly reduced cone responses, but no signs of the rod dysfunction seen in CSNB1. Specifically, the rod response and bright-flash cone-rod mixed ERG amplitudes were normal (Table 1, Figure 3). The patterns of his ERG responses are completely different from CSNB1 patients with NYX mutations [28], but are similar to those of patients with X-linked recessive high myopia [12]. His parents had neither myopia nor CSNB.

**Table 1:** Rod and cone responses of II:1 on ERG recordings.

Responses	Amplitude (μV)		Normal range
	OD	OS	(95% CI)
Rod b-wave	257	275	175-471
Maximum a-wave	275	289	275-481
Maximum b-wave	444	469	414-778
Cone a-wave	23.9	24.3	45-113
Cone b-wave	94.3	91.1	99-237

Patient II:1 in family B had normal rod responses and mildly reduced cone responses.

Both the c.144C>G and c.572-573GC>AA mutations would result in missense changes of the encoded amino acids, Cys48Trp and Arg191Gln. These two amino acids are conserved between human and mouse NYX [37] but are not conserved in human chondroadherin, the closest member of the small leucine rich protein (SLRP) gene family [18]. The Cys48Trp mutation is a severe change at protein level with a residue weight of -2 (Blosum80). The Arg191Gln mutation, a Blosum80 score of +1 for this substitution, changed a charged residue to a polar residue with otherwise similar properties. These two mutations were not detected in 232 controls individuals of Southern Chinese ethnic origin (totally 324 X chromosome from 140 males and 92 females). Neither were they detected in previous studies nor present in the human NYX SNP database, nor have other mutations at Cys48 or Arg191 been detected before. The mutations are unlikely to be random occurrences since no missense changes have previously been identified in the human NYX gene. The sequence of the NYX coding region is very conserved: only three synonymous variations are recorded in the SNP database, including rs12013709, rs11798969, and rs3810733. Of these only rs3810733 was detected in Chinese individuals analyzed in this study. However, a c.1198G>A variation (Gly400Ser with a Blosum 80 score of zero) was detected in 2 of the 52 probands. This variation was also detected in 5 of 232 controls (3 of 140 males and 2 of 92 females) who had neither high myopia nor had stationary night blindness. The Gly400Ser sequence change is the first polymorphism causing an amino acid change so far identified in NYX.

Application of the web-based method for protein fold recognition using 1D and 3D sequence profiles coupled with secondary structure and solvation potential information (3D-PSSM) yielded the Nogo receptor as the best match to the NYX structure (PSSM E-value=0.001222) with a sequence identity for 285 residues of 33%. Therefore, the atomic coordinates of the Nogo receptor (PDB file: 1ozn) were selected as structural templates.

**Figure 4:**
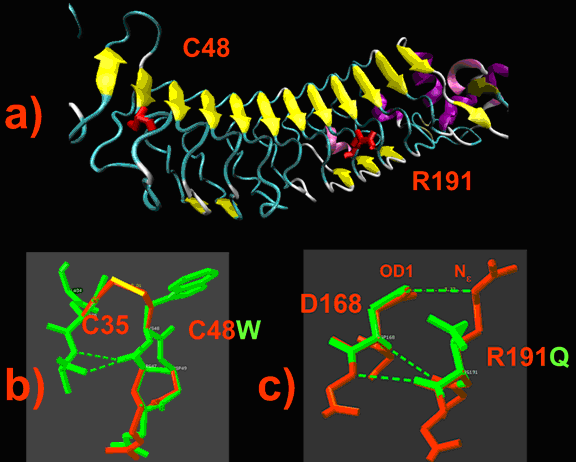
Structural model of nyctalopin built by homology modeling and effect of genetic mutations. **A**: 3-Dimensional structure of nyctalopin. Yellow arrows show beta-strands. The side chains of C48 and R191 are shown in red. **B**: Detail of the region surrounding the C48W mutation showing disruption of disulfide bonds. **C**: Detail of the effect of the R191Q mutation showing loss of hydrogen bonds. The normal and mutated nyctalopin structures are shown in red and green, respectively. Hydrogen and disulphide bonds are shown by green dashed and yellow solid lines, respectively.

Nyctalopin adopts a leucine-rich repeat (LRR) module with concave exterior surface containing short patches of aromatic and arginine residues, as does the Nogo receptor (Figure 4A). The mutations Cys48Trp and Arg191Gln were analyzed based on homology modeling atomic models of nyctalopin as described in the Methods section, and their locations are shown in Figure 4A. The Cys48Trp mutation changes cysteine 48 to a hydrophobic tryptophan and will break the disulphide bridge between Cys48 and Cys35, which stabilizes the structure of the beta-finger located in the N-terminal cap domain (Figure 4B). Arginine 191 is located on the surface of nyctalopin and forms the inter-side chain hydrogen bond between the Ne and OD1 atoms of Arg191 and Asp168, respectively (Figure 4C). Changing Argenine 191 to Glutamine will break this hydrogen bond and might be expected to disrupt the concave exterior surface of the molecule. While this analysis is not definitive, it does suggest that both the Cys48Trp and Arg191Gln mutations are likely to destabilize specific parts of nyctalopin significantly without resulting in a complete loss of structure.

**Figure 5:**
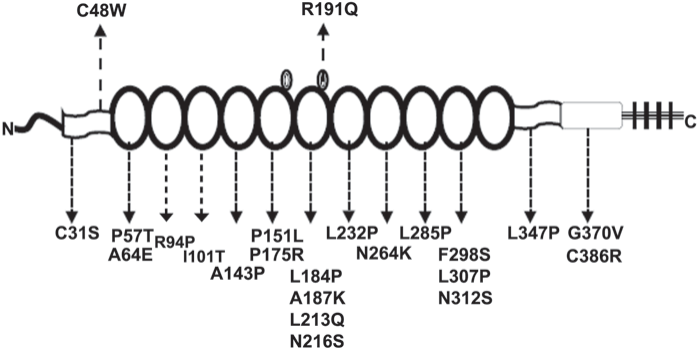
Diagram of nyctalopin with missense mutations identified so far (Other mutations causing null alleles were not listed here). Nyctalopin has an NH_2_-terminal signal sequence, NH_2_-terminal cysteine cluster, 11 central LRRs (circles), a COOH-terminal cluster, a region before GPI, and a GPI-anchor site. Two small circles are put on LRR5 and LRR6, indicating the two small loops inside the nyctalopia as suggested [18]. The two mutations described here are shown on the top of the protein diagram and those mutations previously reported on the bottom.

The Arg191Gln mutation is situated in a position different from all the missense mutations known to cause CSNB1 (Figure 5). This mutation is located within the sixth repeat of leucine-rich repeat in NYX (LRR6), one of two loops carrying additional amino acid sequences [18]. To date, the functional property of these two modified loops is not known but mutations at these additional sequences have not been previously identified. The Cys48Trp mutation occurs in the last cysteine of the N-terminal cysteine cluster, Cx3Cx3CxCx6Cx3C [18]. A missense mutation at the second cysteine of this cluster, Cys31Ser, resulted in CSNB1 [19]. Current knowledge of nyctalopin is not sufficient to provide an explanation of the different phenotypes resulting from the Cys31Ser and Cys48Trp mutations. However, different mutations in a single gene causing such varied phenotypes as progressive retinitis pigmentosa or stationary night blindness have been described previously, including PDE6B (OMIM 180072), RHO (OMIM 180380), and SAG (OMIM 181031). The high myopia seen in CSNB1 is gene-specific rather than associated with night blindness in general. Night blindness, progressive or stationary, has been observed in a number of diseases, but high myopia is not always associated with such diseases except for those resulted from mutations in RP2 and NYX.

Retinal electrophysiology studies have revealed that the functional defects in CSNB1 involve retinal ON-pathways of rod and cone signals [38]. Such abnormal ON-responses were also observed in congenital stationary night blindness associated with mutations in the GRM6 gene [39]. It is interesting that two of the three patients with GRM6 mutations had moderate myopia and one individual with two mutations in one GRM6 allele had high myopia [39]. There are two parallel ON-pathways in response to rod or cone signals [40]. While the above correlations are certainly not definitive evidence, they do suggest that aberrations in the cone signal resulting from mutations in GRM6 and NYX might participate in myopia formation. It has been demonstrated that blurred visual images can induce myopia [1,41,42]. Such images might initially create a "noisy" or aberrant retinal signal before the local eye or central nervous system can react to the retinal blur. Similarly, variations in proteins acting in signal transmission might also create a "noisy" or aberrant retinal signal mimicking the effects of retinal blur and therefore induce myopia formation. While local changes in metabolic conditions or scleral growth and development seem likely to contribute directly, further analysis of the retinal ON-pathway for non-syndromic high myopia, whether transmitted autosomally or as an X-linked trait, might provide additional clues for understanding the molecular basis of myopia.

In summary, the results presented here suggest that mutations in the NYX gene may cause isolated high myopia without associated night blindness. This further suggests that disturbances in the retinal ON-pathway might lead to myopia and provides a useful clue in the search for genes responsible for high myopia as well as elucidating additional functional properties of nyctalopin.
